# Biomarkers for IgA nephropathy on the basis of multi-hit pathogenesis

**DOI:** 10.1007/s10157-018-1582-2

**Published:** 2018-05-08

**Authors:** Hitoshi Suzuki

**Affiliations:** Department of Nephrology, Juntendo University Faculty of Medicine, Tokyo, Japan

**Keywords:** IgA nephropathy, Galactose-deficient IgA1, Immune complexes, Biomarker

## Abstract

IgA nephropathy (IgAN) is the most prevalent glomerular disease worldwide and is associated with a poor prognosis. Development of curative treatment strategies and approaches for early diagnosis is necessary. Renal biopsy is the gold standard for the diagnosis and assessment of disease activity. However, reliable biomarkers are needed for the noninvasive diagnosis of this disease and to more fully delineate the risk of progression. With regard to the pathogenesis of IgAN, the multi-hit hypothesis, including production of galactose-deficient IgA1 (Gd-IgA1; Hit 1), IgG or IgA autoantibodies that recognize Gd-IgA1 (Hit 2), and their subsequent immune complexes formation (Hit 3) and glomerular deposition (Hit 4), has been widely supported by many studies. Although the prognostic values of several biomarkers have been discussed, we recently developed a highly sensitive and specific diagnostic method by measuring serum levels of Gd-IgA1 and Gd-IgA1-containing immune complexes. In addition, urinary Gd-IgA1 may represent a disease-specific biomarker for IgAN. We also confirmed that there is a significant correlation between serum levels of these effector molecules and disease activity, suggesting that each can be considered a practical surrogate marker of therapeutic response. Thus, these disease-oriented specific serum and urine biomarkers may be useful for screening of potential IgAN with isolated hematuria, earlier diagnosis, disease activity, and eventually, response to treatment. In this review, we discuss these concepts, with a focus on potential clinical applications of these biomarkers.

## Introduction

IgA nephropathy (IgAN) is the most common form of glomerular disease worldwide and is associated with a poor prognosis, resulting in end-stage kidney disease (ESKD) in approximately 40% of cases within 20–30 years [1–3]. Patients can present with a range of signs and symptoms, from asymptomatic microscopic haematuria to macroscopic haematuria and/or proteinuria. Currently, no IgAN-specific therapies are available; therefore, development of a curative treatment and strategies for early diagnosis and treatment are urgently needed. Although the prognostic and predictive values of several markers have been discussed elsewhere, we recently developed a highly sensitive and specific diagnostic method as well as assessment of disease activity and prognosis by measuring serum levels of aberrantly glycosylated serum IgA1 and related IgA immune complexes. In this review, we focus on potential clinical applications of these biomarkers.

## Limitations of renal biopsy and proteinuria for assessment of disease activity

The poor prognosis of IgAN is partly due to delayed diagnosis. Renal biopsy is the gold standard for diagnosis as well as assessment of disease activity and prognosis in patients with IgAN. However, the pathological findings differ according to the time point of renal biopsy during the long clinical course of IgAN [1, 2]. Different timing of intervention for renal biopsy may yield variations in pathological severity and chronicity on renal biopsy. Moreover, renal biopsy is not frequently performed because of procedural risks and/or limitations of medical insurance coverage. In fact, renal biopsy is not recommended for patients with isolated hematuria or mild proteinuria in Western countries, where renal biopsy is performed for those who develop increasing proteinuria or worsening renal function [4].

Thus, renal biopsy provides information regarding transient conditions and has limited ability to provide an accurate assessment of disease activity. Furthermore, even if biopsy is performed during early stages of IgAN, pathological findings may be inconclusive, and it may be difficult to establish a prognosis. Moreover, in patients with IgAN accompanied by mild proteinuria with mild histological lesions at the time of renal biopsy, progression of proteinuria is observed in approximately 30–40% of cases [3, 5, 6]. Thus, novel noninvasive biomarkers are needed for evaluation of real-time disease activity.

The degree of proteinuria is an important prognostic factor in IgAN and other renal diseases [7, 8], and many clinical studies have reported analysis of kidney function and proteinuria as endpoints in renal disease [9, 10]. Therefore, many clinical guidelines recommend therapeutic indication based on the degree of proteinuria [11, 12]. However, it is difficult to distinguish proteinuria from acute glomerular inflammatory lesions, such as cellular crescents or burned-out sclerotic glomerulus, in patients with IgAN. In general, IgAN may have a long chronic course with a mixture of acute inflammatory lesions and common pathway-based chronic lesions [13], and proteinuria must be derived from both types of lesions. Accordingly, it is difficult to qualitatively discriminate proteinuria at the acute and chronic phases, and proteinuria-based assessment of disease activity is limited.

Based on this background, activity assessment methods other than renal biopsy and urinalysis are needed. In addition to assessment of disease activity, a simple and safe method for early diagnosis using valid biomarkers based on the pathogenesis of IgAN should be established.

## Pathogenic importance of aberrantly glycosylated IgA1 in IgAN

**Fig. 1:**
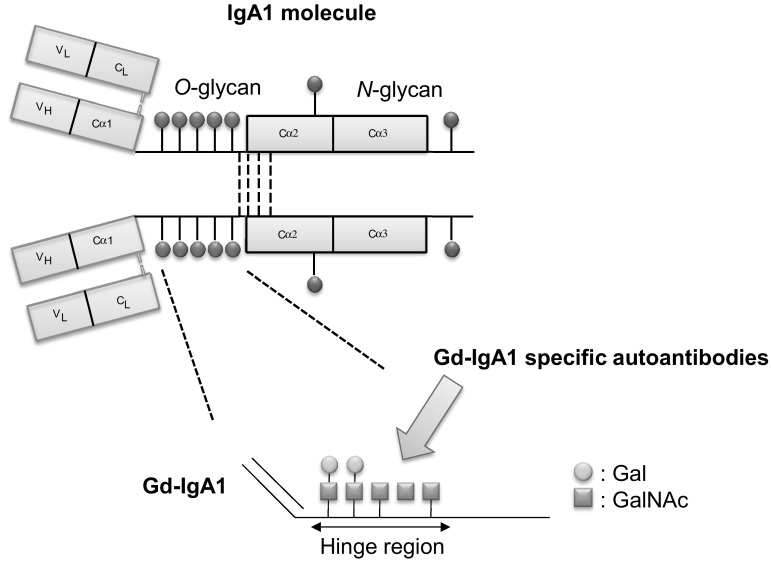
Structure of IgA1 and synthesis of IgA1 *O*-glycans. IgA1 has *O*-glycans located in the unique hinge region between constant-region domains 1 and 2 of the heavy chain. Serum levels of IgA1 with galactose-deficient glycans (Gd-IgA1) are elevated in patients with IgA nephropathy. The *Helix aspersa* agglutinin lectin and Gd-IgA1-specific autoantibodies recognize the galactose-deficient GalNAc

IgA in glomerular deposits is exclusively of the IgA1 subclass, and levels of the polymeric form of IgA1 are elevated in the serum of patients with IgAN [14–16]. Galactose deficiency of *O*-linked glycans in the hinge region of IgA1 is the beginning of a sequence of events that may lead to renal injuries. This galactose-deficient IgA1 (Gd-IgA1) consists of terminal *N*-acetylgalactosamine (GalNAc) or sialylated GalNAc [17–19]. Normal serum IgA1 is thought to contain little or no galactose-deficient *O*-glycans [20] (Fig. 1), but some *O*-glycans of circulatory IgA1 in healthy individuals are galactose-deficient [21]. In recent years, an increase in serum Gd-IgA1 levels in patients with IgAN was quantitatively confirmed for the first time by Moldoveanu et al. using *Helix aspersa* agglutinin lectin, which recognizes GalNAc residues [22]. Analysis of immortalized IgA1-secreting cells derived from the circulation of patients with IgAN and healthy controls has shown that Gd-IgA1 is related to decreased activity of core 1 β1,3-galactosyltransferase (C1GalT1) and elevated activity of α-2,6-sialyltransferase 2 (ST6GalNAc-II) [23]. Recent studies have shown that these enzymatic activities may be regulated by genetic mechanisms and dysregulation of mucosal immunity [24–26]. Mucosal infections, such as tonsillitis and upper respiratory infections, are associated with exacerbation of urinary abnormalities in patients with IgAN. In fact, Gd-IgA1-dependent modulation of C1GalT1 and ST6GalNAc-II is induced by interleukin (IL)-6 and IL-4 [26]. Importantly, Toll-like receptor (TLR) 9 plays a key role in the progression of IgAN [27, 28], and overexpression of tonsillar TLR9 is correlated with the production of Gd-IgA1 [29]. Furthermore, repeated TLR9 activation induces tonsillar expression of a proliferation-inducing ligand (April), which participates in the generation and survival of antibody-producing plasma cells, resulted in production of Gd-IgA1 [30].

Previous reports have indicated that glomerular IgA1 in IgAN is aberrantly glycosylated [31, 32]. A fraction of Gd-IgA1 from the glomerular deposits is excreted into the urine and thus represents a disease-specific marker of IgAN. Urinary excretion of Gd-IgA1 discriminates patients with IgAN from patients with other proteinuric renal diseases [33]. Furthermore, the level of urinary Gd-IgA1 is correlated with proteinuria in patients with IgAN. Urinary Gd-IgA1 thus may represent a disease-specific marker of IgAN. We recently established a novel lectin-independent method exploiting monoclonal antibody (KM55 mAb) for measuring serum levels of Gd-IgA1 [34]; this method could be used worldwide for standardized measurement of serum Gd-IgA1 (Immuno-Biological Laboratories Co., Ltd.). In addition, we verified glomerular Gd-IgA1 was specifically detected in IgAN [35]. Further studies are ongoing to establish a measurement system for urinary Gd-IgA1 using KM55 mAb.

## Essential role of Gd-IgA1-specific antibodies

**Fig. 2:**
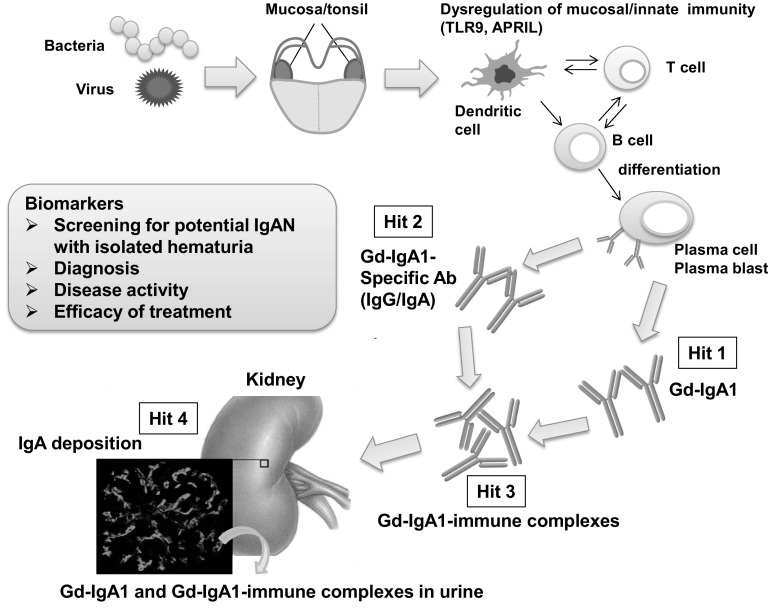
Hypothesis for the pathogenesis of IgA nephropathy. Synthesis of IgA1 with some *O*-glycans deficient in galactose (autoantigen) is elevated. Gd-IgA1 is present in the circulation at increased levels (Hit 1). This immunoglobulin is recognized by unique circulating anti-glycan autoantibodies (Hit 2). This process results in the formation of pathogenic IgA1-containing circulating immune complexes (Hit 3), some of which deposit in the glomeruli and induce renal injury (Hit 4). Upstream factors are likely involved in abnormal mucosal/innate immune responses characteristic for patients with IgA nephropathy

There is an increasing evidence that Gd-IgA1 has a pivotal role in the pathogenesis of IgAN [36, 37]. Serum Gd-IgA1 levels in relatives were elevated compared with those in normal individuals who were not blood relatives, regardless of the absence of nephropathy [38]. In vitro, Gd-IgA1-containing immune complexes, but not Gd-IgA1 alone, induces the proliferation of mesangial cells [39]. Furthermore, the concentration of immune complexes containing Gd-IgA1 is increased in the blood and urine of patients with IgAN [40–42]. Thus, these facts implied that additional pathogenic hits are necessary in the pathogenesis of IgAN. Gd-IgA1 in the serum of patients with IgAN is found exclusively within immune complexes bound to IgG or IgA1 antibodies. IgG autoantibodies recognize glycan-containing epitopes on Gd-IgA1 (Fig. 1) and exhibit unique features in the complementarity-determining region 3 of the variable region of their heavy chains [43]. Furthermore, serum levels of Gd-IgA1-specific IgG autoantibodies are correlated with disease severity, as assessed by the magnitude of proteinuria [43]. The onset and progression of IgAN is believed to require Gd-IgA1 (Hit 1), as well as endogenous anti-glycan antibodies (Hit 2) and subsequent immune complexes formation (Hit 3) and glomerular deposition (Hit 4; Fig. 2) [36].

## Gd-IgA1 and its related immune complexes as biomarkers for disease activity of IgAN

The above-described clinical findings suggested that Gd-IgA1 and its related immune complexes containing anti-glycan autoantibodies are essential effector molecules in pathogenesis of IgAN. Recent studies have demonstrated that increased serum Gd-IgA1 levels are associated with exacerbation of proteinuria and a greater risk of deterioration of renal function in IgAN [44]. In addition, the combination of high serum Gd-IgA1 levels and circulating levels of advanced oxidation protein products is correlated with a more rapid decline in estimated glomerular filtration rate, suggesting that oxidative stress linked to Gd-IgA1 may be involved in the pathogenesis of IgAN [45]. Although these reports did not analyze serum levels of IgA1-containing immune complexes, the serum levels of Gd-IgA1-specific IgG autoantibodies have been found to be correlated with disease severity [43]. Furthermore, Berthoux et al. reported that serum levels of Gd-IgA1-specific IgG and IgA autoantibodies at the time of biopsy are significantly associated with progression of IgAN towards dialysis or death [46]. Although a prognostic biomarker for recurrence after renal transplant is lacking in patients with IgAN, Gd-IgA1-specific IgG autoantibody level is associated with higher risk of recurrence [47]. These findings further confirmed the multi-hit hypothesis as the pathogenic model of IgAN [36] and indicated that evaluation of serum levels of the autoantigen (Gd-IgA1) and autoantibodies (IgG or IgA subclass) should be required as biomarkers of IgAN.

To evaluate therapeutic efficacy, we measured changes in serum levels of Gd-IgA1 before and after tonsillectomy. Cases with IgAN who showed significant decreases in serum Gd-IgA1 levels after tonsillectomy achieved significantly better improvement in hematuria [29]. Moreover, serum levels of Gd-IgA1 and IgA-IgG IC were examined before and after tonsillectomy with steroid pulse therapy [48]. Cross-sectional analysis revealed that the amounts of hematuria and proteinuria were significantly associated with serum levels of Gd-IgA1 and levels of IgA-IgG immune complexes [48]. These non-invasive biomarkers for disease activity may be useful for guiding therapeutic approaches in the future.

## Gd-IgA1 and its related immune complexes as biomarkers for the diagnosis of IgAN

Moldoveanu et al. first investigated the quantification of serum levels of Gd-IgA1 as a diagnostic test [22]. By receiver operating characteristic curve analysis, the serum level of Gd-IgA1 that provided 0.77 sensitivity had a specificity of 0.90 to distinguish patients with IgAN from healthy controls. We recently reported that serum levels of IgA, Gd-IgA1, Gd-IgA1-specific IgG, and Gd-IgA1-specific IgA were elevated in patients with IgAN compared with those of healthy controls and patients with other renal diseases [49]. Gd-IgA1-specific IgG showed the best performance for the diagnosis of IgAN, with a sensitivity of 89% and specificity of 92%. Although those biomarkers may be useful for the diagnosis of IgAN, there is substantial overlap in serum levels of individual biomarkers between patients with IgAN, other renal diseases, and healthy controls [49]. Consequently, no single biomarker was sufficiently specific for IgAN. These data suggested that a panel of such serum biomarkers may be helpful to differentiate IgAN from other glomerular diseases. In addition, other related markers, such as urinary Gd-IgA1 and IgG-IgA immune complexes, may be better to include in larger cohorts. Importantly, a multicenter trial of the diagnostic use of the panel of biomarkers has already been initiated in Japan.

## Gd-IgA1 and its related immune complexes as biomarkers for early screening of IgAN

In Japan, annual checkups, including urine analysis, are performed beginning in childhood. About 75% of Japanese patients with IgAN are initially identified through chance hematuria. Although hematuria must be an initial manifestation of IgAN, glomerular injury leading to hematuria may precede that leading to persistent proteinuria. Thus, epidemiological studies assessing risk factors for chronic kidney disease have indicated that hematuria is a risk factor for proteinuria [7, 50]. Renal biopsy is not recommended for patients presenting with isolated hematuria. Early diagnosis and early intervention are essential to increase the chance of clinical remission [51]. However, there are still many patients who show delayed intervention of therapy, resulting in deterioration of renal function. Therefore, these biomarkers may be applicable for secondary screening of examinees with hematuria in general checkups. Multicenter trials of early screening for patients with hematuria have already been started using a panel of biomarkers in Japan. Hematuria generally occurs prior to proteinuria in IgAN, and new screening systems with these biomarkers may dramatically change the importance of hematuria screening.

## Conclusion

IgAN is the most common form of glomerular disease worldwide and is associated with a poor prognosis. Specific curative treatment strategies are needed for the management of IgAN. Emerging evidence has indicated that Gd-IgA1 and Gd-IgA1-containing immune complexes are essential effector molecules of IgAN. As discussed in this review, recent clinical and experimental studies have emphasized that Gd-IgA1 and Gd-IgA1-specific autoantibodies are prospective biomarkers for diagnostic and disease activity assessment in IgAN. Several clinical studies testing novel medications for IgAN are ongoing worldwide. Noninvasive and real-time examinations with such biomarkers on the basis of pathogenesis, as alternatives to renal biopsy, are critical for determining treatment efficacy and disease activity. Moreover, for the early screening of potential IgAN with isolated hematuria, those biomarkers may be applicable for early intervention and result in reduced risk of deterioration of renal function (Fig. 2).

## References

[1] Chauveau D, Droz D (1993). Follow-up evaluation of the first patients with IgA nephropathy described at Necker Hospital. Contrib Nephrol.

[2] D’Amico G (2004). Natural history of idiopathic IgA nephropathy and factors predictive of disease outcome. Semin Nephrol.

[3] Imai H, Miura N (2012). A treatment dilemma in adult immunoglobulin a nephropathy: what is the appropriate target, preservation of kidney function or induction of clinical remission?. Clin Exp Nephrol.

[4] Donadio JV, Grande JP (2002). IgA Nephropathy. N Engl J Med.

[5] Szeto CC, Lai FM, To KF, Wong TY, Chow KM, Choi PC, Lui SF, Li PK (2001). The natural history of immunoglobulin: a nephropathy among patients with hematuria and minimal proteinuria. Am J Med.

[6] Shen P, He L, Li Y, Wang Y, Chan M (2007). Natural history and prognostic factors of IgA nephropathy presented with isolated microscopic hematuria in Chinese patients. Nephron Clin Pract.

[7] Iseki K, Ikemiya Y, Iseki C, Takishita S (2003). Proteinuria and the risk of developing end-stage renal disease. Kidney Int.

[8] Imai E, Horio M, Yamagata K, Iseki K, Hara S, Ura N, Kiyohara Y, Makino H, Hishida A, Matsuo S (2008). Slower decline of glomerular filtration rate in the Japanese general population: A longitudinal 10-year follow-up study. Hypertens Res.

[9] Pozzi C, Andrulli S, Del Vecchio L, Melis P, Fogazzi GB, Altieri P, Ponticelli C, Locatelli F (2004). Corticosteroid effectiveness in IgA nephropathy: long-term results of a randomized, controlled trial. J Am Soc Nephrol.

[10] Li PK, Leung CB, Chow KM, Cheng YL, Fung SK, Mak SK, Tang AW, Wong TY, Yung CY, Yung JC, Yu AW, Szeto CC, HKVIN Study Group (2006). Hong Kong study using valsartan in IgA nephropathy (HKVIN): a double-blind, randomized, placebo-controlled study. Am J Kidney Dis.

[11] Radhakrishnan J, Cattran DC (2012). The KDIGO practice guideline on glomerulonephritis: reading between the (guide) lines–application to the individual patient. Kidney Int.

[12] Japanese Society of Nephrology (2014). Evidence-based clinical practice guideline for CKD 2013. Clin Exp Nephrol.

[13] D’Amico G (2000). Natural history of idiopathic IgA nephropathy: role of clinical and histological prognostic factors. Am J Kidney Dis.

[14] Conley ME, Cooper MD, Michael AF (1980). Selective deposition of immunoglobulin A1 in immunoglobulin a nephropathy, anaphylactoid purpura nephritis, and systemic lupus erythematosus. J Clin Invest.

[15] Tomino Y, Endoh M, Nomoto Y, Sakai H (1981). Immunoglobulin A1 and IgA nephropathy. N Engl J Med.

[16] Barratt J, Feehally J, Smith AC (2004). Pathogenesis of IgA nephropathy. Semin Nephrol.

[17] Allen AC, Harper SJ, Feehally J (1995). Galactosylation of *N*- and *O*-linked carbohydrate moieties of IgA1 and IgG in IgA nephropathy. Clin Exp Immunol.

[18] Mestecky J, Tomana M, Crowley-Nowick PA, Moldoveanu Z, Julian BA, Jackson S (1993). Defective galactosylation and clearance of IgA1 molecules as a possible etiopathogenic factor in IgA nephropathy. Contrib Nephrol.

[19] Tomana M, Matousovic K, Julian BA, Radl J, Konecny K, Mestecky J (1997). Galactose-deficient IgA1 in sera of IgA nephropathy patients is present in complexes with IgG. Kidney Int.

[20] Mattu TS, Pleass RJ, Willis AC, Kilian M, Wormald MR, Lellouch AC, Rudd PM, Woof JM, Dwek RA (1998). The glycosylation and structure of human serum IgA1, Fab, and Fc regions and the role of *N*-glycosylation on Fcα receptor interactions. J Biol Chem.

[21] Renfrow MB, Cooper HJ, Tomana M, Kulhavy R, Hiki Y, Toma K, Emmett MR, Mestecky J, Marshall AG, Novak J (2005). Determination of aberrant *O*-glycosylation in the IgA1 hinge region by electron capture dissociation fourier transform-ion cyclotron resonance mass spectrometry. J Biol Chem.

[22] Moldoveanu Z, Wyatt RJ, Lee JY, Tomana M, Julian BA, Mestecky J, Huang WQ, Anreddy SR, Hall S, Hastings MC, Lau KK, Cook WJ, Novak J (2007). Patients with IgA nephropathy have increased serum galactose-deficient IgA1 levels. Kidney Int.

[23] Raska M, Moldoveanu Z, Suzuki H, Brown R, Kulhavy R, Andrasi J, Hall S, Vu HL, Carlsson F, Lindahl G, Tomana M, Julian BA, Wyatt RJ, Mestecky J, Novak J (2007). Identification and characterization of CMP-NeuAc:GalNAc-IgA1 α2,6-sialyltransferase in IgA1-producing cells. J Mol Biol.

[24] Kiryluk K, Li Y, Sanna-Cherchi S, Rohanizadegan M, Suzuki H, Eitner F, Snyder HJ, Choi M, Hou P, Scolari F, Izzi C, Gigante M, Gesualdo L, Savoldi S, Amoroso A, Cusi D, Zamboli P, Julian BA, Novak J, Wyatt RJ, Mucha K, Perola M, Kristiansson K, Viktorin A, Magnusson PK, Thorleifsson G, Thorsteinsdottir U, Stefansson K, Boland A, Metzger M, Thibaudin L, Wanner C, Jager KJ, Goto S, Maixnerova D, Karnib HH, Nagy J, Panzer U, Xie J, Chen N, Tesar V, Narita I, Berthoux F, Floege J, Stengel B, Zhang H, Lifton RP, Gharavi AG (2012). Geographic differences in genetic susceptibility to IgA nephropathy: GWAS replication study and geospatial risk analysis. PLoS Genet.

[25] Kiryluk K, Novak J (2014). The genetics and immunobiology of IgA nephropathy. J Clin Invest.

[26] Suzuki H, Raska M, Yamada K, Moldoveanu Z, Julian BA, Wyatt RJ, Tomino Y, Gharavi AG, Novak J (2014). Cytokines alter IgA1 *O*-glycosylation by dysregulating C1GalT1 and ST6GalNAc-II enzymes. J Biol Chem.

[27] Suzuki H, Suzuki Y, Narita I, Aizawa M, Kihara M, Yamanaka T, Kanou T, Tsukaguchi H, Novak J, Horikoshi S, Tomino Y (2008). Toll-like receptor 9 affects severity of IgA nephropathy. J Am Soc Nephrol.

[28] Sato D, Suzuki Y, Kano T, Suzuki H, Matsuoka J, Yokoi H, Horikoshi S, Ikeda K, Tomino Y (2012). Tonsillar TLR9 expression and efficacy of tonsillectomy with steroid pulse therapy in IgA nephropathy patients. Nephrol Dial Transpl.

[29] Nakata J, Suzuki Y, Suzuki H, Sato D, Kano T, Yanagawa H, Matsuzaki K, Horikoshi S, Novak J, Tomino Y (2014). Changes in nephritogenic serum galactose-deficient IgA1 in IgA nephropathy following tonsillectomy and steroid therapy. PLoS One.

[30] Muto M, Manfroi B, Suzuki H, Joh K, Nagai M, Wakai S, Righini C, Maiguma M, Izui S, Tomino Y, Huard B, Suzuki Y (2017). Toll-like receptor 9 stimulation induces Aberrant expression of a proliferation-inducing Ligand by Tonsillar germinal center B cells in IgA nephropathy. J Am Soc Nephrol.

[31] Hiki Y, Odani H, Takahashi M (2001). Mass spectrometry proves under-O-glycosylation of glomerular IgA1 in IgA nephropathy. Kidney Int.

[32] Allen AC, Bailey EM, Brenchley PE, Buck KS, Barratt J, Feehally J (2001). Mesangial IgA1 in IgA nephropathy exhibits aberrant O-glycosylation: observations in three patients. Kidney Int.

[33] Suzuki H, Allegri L, Suzuki Y, Hall S, Moldoveanu Z, Wyatt RJ, Novak J, Julian BA. Galactose-deficient IgA1 as a candidate urinary polypeptide marker of IgA nephropathy? Dis Mark 7806438, 2016. 10.1155/2016/7806438 PMC5018335 27647947

[34] Yasutake J, Suzuki Y, Suzuki H, Hiura N, Yanagawa H, Makita Y, Kaneko E, Tomino Y. Novel lectin-independent approach to detect galactose-deficient IgA1 in IgA nephropathy 30: 1315–21, 2015. 10.1093/ndt/gfv221 PMC4513896 26109484

[35] Suzuki H, Yasutake J, Makita Y, Tanbo Y, Yamazaki K, Sofue T, Kano T, Suzuki Y (2018). IgA nephropathy and IgA vasculitis with nephritis have a shared feature involving galactose-deficient IgA1 oriented pathogenesis. Kidney Int.

[36] Suzuki H, Kiryluk K, Novak J, Moldoveanu Z, Herr AB, Renfrow MB, Wyatt RJ, Scolari F, Mestecky J, Gharavi AG, Julian BA (2011). The pathophysiology of IgA nephropathy. J Am Soc Nephrol.

[37] Glassock RJ (2011). The pathogenesis of IgA nephropathy. Curr Opin Nephrol Hypertens.

[38] Gharavi AG, Moldoveanu Z, Wyatt RJ, Barker CV, Woodford SY, Lifton RP, Mestecky J, Novak J, Julian BA (2008). Aberrant IgA1 glycosylation is inherited in familial and sporadic IgA nephropathy. J Am Soc Nephrol.

[39] Novak J, Tomana M, Matousovic K, Brown R, Hall S, Novak L, Julian BA, Wyatt RJ, Mestecky J (2005). IgA1-containing immune complexes in IgA nephropathy differentially affect proliferation of mesangial cells. Kidney Int.

[40] Novak J, Julian BA, Mestecky J, Renfrow MB (2012). Glycosylation of IgA1 and pathogenesis of IgA nephropathy. Semin Immunopathol.

[41] Mestecky J, Raska M, Julian BA, Gharavi AG, Renfrow MB, Moldoveanu Z, Novak L, Matousovic K, Novak J (2013). IgA nephropathy: molecular mechanisms of the disease. Annu Rev Pathol.

[42] Matousovic K, Novak J, Yanagihara T, Tomana M, Moldoveanu Z, Kulhavy R, Julian BA, Konecny K, Mestecky J (2006). IgA-containing immune complexes in the urine of IgA nephropathy patients. Nephrol Dial Transpl.

[43] Suzuki H, Fan R, Zhang Z, Brown R, Hall S, Julian BA, Chatham WW, Suzuki Y, Wyatt RJ, Moldoveanu Z, Lee JY, Robinson J, Tomana M, Tomino Y, Mestecky J, Novak J (2009). Aberrantly glycosylated IgA1 in IgA nephropathy patients is recognized by IgG antibodies with restricted heterogeneity. J Clin Invest.

[44] Zhao N, Hou P, Lv J, Moldoveanu Z, Li Y, Kiryluk K, Gharavi AG, Novak J, Zhang H (2012). The level of galactose-deficient IgA1 in the sera of patients with IgA nephropathy is associated with disease progression. Kidney Int.

[45] Camilla R, Suzuki H, Daprà V, Loiacono E, Peruzzi L, Amore A, Ghiggeri GM, Mazzucco G, Scolari F, Gharavi AG, Appel GB, Troyanov S, Novak J, Julian BA, Coppo R (2011). Oxidative stress and galactose-deficient IgA1 as markers of progression in IgA nephropathy. Clin J Am Soc Nephrol.

[46] Berthoux F, Suzuki H, Thibaudin L, Yanagawa H, Maillard N, Mariat C, Tomino Y, Julian BA, Novak J (2012). Autoantibodies targeting galactose-deficient IgA1 associate with progression of IgA nephropathy. J Am Soc Nephrol.

[47] Berthoux F, Suzuki H, Mohey H, Maillard N, Mariat C, Novak J, Julian BA (2017). Prognostic value of serum biomarkers of autoimmunity for recurrence of IgA nephropathy after kidney transplantation. J Am Soc Nephrol.

[48] Suzuki Y, Matsuzaki K, Suzuki H, Okazaki K, Yanagawa H, Ieiri N, Sato M, Sato T, Taguma Y, Matsuoka J, Horikoshi S, Novak J, Hotta O, Tomino Y (2014). Serum levels of galactose-deficient immunoglobulin (Ig) A1 and related immune complex are associated with disease activity of IgA nephropathy. Clin Exp Nephrol.

[49] Yanagawa H, Suzuki H, Suzuki Y, Kiryluk K, Gharavi AG, Matsuoka K, Makita Y, Julian BA, Novak J, Tomino Y (2014). A panel of serum biomarkers differentiates IgA nephropathy from other renal diseases. PLoS One.

[50] Yamagata K, Ishida K, Sairenchi T, Takahashi H, Ohba S, Shiigai T, Narita M, Koyama A (2007). Risk factors for chronic kidney disease in a community-based population: a 10-year follow-up study. Kidney Int.

[51] Ieiri N, Hotta O, Sato T, Taguma Y (2012). Significance of the duration of nephropathy for achieving clinical remission in patients with IgA nephropathy treated by tonsillectomy and steroid pulse therapy. Clin Exp Nephrol.

